# Casanovas are liars: behavioral syndromes, sperm competition risk, and the evolution of deceptive male mating behavior in live-bearing fishes

**DOI:** 10.12688/f1000research.2-75.v3

**Published:** 2013-10-23

**Authors:** David Bierbach, Amber M Makowicz, Ingo Schlupp, Holger Geupel, Bruno Streit, Martin Plath

**Affiliations:** 1 Evolutionary Ecology Group, University of Frankfurt, Frankfurt am Main, D-60438, Germany; 2 Current address: Biology and Ecology of Fishes, Leibniz-Institute of Freshwater Ecology and Inland Fisheries, Berlin, Germany; 3 Department of Biology, University of Oklahoma, Norman, OK 73019, USA

## Abstract

Male reproductive biology can by characterized through competition over mates as well as mate choice. Multiple mating and male mate choice copying, especially in internally fertilizing species, set the stage for increased sperm competition, i.e., sperm of two or more males can compete for fertilization of the female’s ova. In the internally fertilizing fish *Poecilia mexicana*, males respond to the presence of rivals with reduced expression of mating preferences (audience effect), thereby lowering the risk of by-standing rivals copying their mate choice. Also, males interact initially more with a non-preferred female when observed by a rival, which has been interpreted in previous studies as a strategy to mislead rivals, again reducing sperm competition risk (SCR). Nevertheless, species might differ consistently in their expression of aggressive and reproductive behaviors, possibly due to varying levels of SCR. In the current study, we present a unique data set comprising ten poeciliid species (in two cases including multiple populations) and ask whether species can be characterized through consistent differences in the expression of aggression, sexual activity and changes in mate choice under increased SCR. We found consistent species-specific differences in aggressive behavior, sexual activity as well as in the level of misleading behavior, while decreased preference expression under increased SCR was a general feature of all but one species examined. Furthermore, mean sexual activity correlated positively with the occurrence of potentially misleading behavior. An alternative explanation for audience effects would be that males attempt to avoid aggressive encounters, which would predict stronger audience effects in more aggressive species. We demonstrate a positive correlation between mean aggressiveness and sexual activity (suggesting a hormonal link as a mechanistic explanation), but did not detect a correlation between aggressiveness and audience effects. Suites of correlated behavioral tendencies are termed behavioral syndromes, and our present study provides correlational evidence for the evolutionary significance of SCR in shaping a behavioral syndrome at the species level across poeciliid taxa.

## Introduction

Female mate choice and male competition are widely acknowledged as the principal forces of sexual selection ^1, 2^, while male mate choice has received comparatively little attention (but see ^3– 5^). Over the past decades, however, it has become apparent that males also express mating preferences ^3, 6– 12^, especially if females show pronounced differences in mate quality ( *e.g*., through size–fecundity relationships ^13^). Nonetheless, male reproductive biology is clearly influenced by competition over mates ^1, 14– 16^, and, at least in species in which females tend to mate with multiple males, this competition extends well into the period after a successful copulation, as sperm of several males can compete for fertilization of the female’s ova ^17– 19^. However, the level of male competition, male mate choice and behavioral responses to perceived sperm competition risk (SCR), may vary between taxa ^20– 22^. An interesting group to study interspecific variation in male aggressive and reproductive behavior is the family Poeciliidae (livebearing fishes), which comprises at least 260 species ^23^. Several members of this family are model organisms for a range of topics in behavior, ecology and evolution ^24^. Nonetheless, comparative approaches in this group mostly considered morphological or physiological traits ^25, 26^, while comparisons of behavioral traits are usually limited to population-level differences (guppy, *Poecilia reticulata*: ^27^), or to a few species commonly used in scientific laboratories ^28, 29^ for exceptions see Dugatkin *et al.* ^30^, and Westneat *et al.* ^31^. Our present study compared ten different species (13 populations) of poeciliid fishes and thus, provides comprehensive insights into potential interspecific variation in male aggressive and reproductive behavior within the family Poeciliidae. Beside aggressiveness and sexual activity, we particularly focused on the presumed role SCR plays for males of this family ^22^.

Theory predicts that males should adjust their mating behavior strategically to imminent SCR ^19, 32^, and several studies on species exhibiting frequent multiple mating confirm that perceived SCR affects male mate choice behavior ^10, 11, 18, 33– 35^. In the Atlantic molly, *Poecilia mexicana*, for instance, males temporarily decrease their sexual activity and cease showing mating preferences when another male is eavesdropping ^9, 18, 21, 36, 37^. It has been hypothesized that those audience-induced changes in male mating behavior prevent rivals from copying mate choice decisions ^19, 32^. Moreover, males initially interact more with a previously non-preferred female in the presence of a rival, which has again been interpreted in the context of mate choice copying — and ultimately, SCR — as males could thus lead the copying male away from the preferred mate (“deceptive mating behavior”; ^21, 36, 38^).

Theoretical considerations identify avoidance of aggressive interactions as another potential mechanism explaining audience-induced changes in male mating behavior ^32^. Specifically, if different males share intrinsic mating preferences ( *e.g.*, for large female body size ^8, 21^), males could interact more equally with different females to reduce the risk of injuries resulting from aggressive interactions over commonly preferred female phenotypes ^32^. If avoiding aggression plays a role, then the magnitude of audience-induced changes in male mating behavior (at the species level) should correlate positively with mean aggressiveness. To test this hypothesis, we examined the intensity of aggressive interactions in size-matched dyadic (paired) male combats for the set of poeciliid species included herein and in an independent approach quantified audience-induced changes of male mate choice in response to an audience (see above) for the same taxa.

Consistency in the expression of a certain behavioral type across different environmental contexts at the inter- *individual* level has received considerable scientific interest ^39– 42^, and suites of correlated behavioral types have been termed behavioral syndromes ^39, 43^. Réale *et al.* ^44^ proposed five different axes of animal personality: shyness–boldness, exploration–avoidance, general activity, aggressiveness, and sociability. Conrad *et al.* ^43^ highlighted several correlations of those behavioral axes in teleost fishes, but audience-induced changes in male mating behavior have not yet been investigated in the context of behavioral syndromes. Recent studies exemplified the importance of population differences in behavioral syndromes ^45, 46^, and the concept of behavioral syndromes was expanded to the comparison of groups of animals or populations. Chapman *et al.* ^47^, for example, demonstrated correlations between mean colony (and caste) behavioral types in *Myrmica* ants. Here, we apply this concept to the comparison of different poeciliid taxa, thus evaluating species-specific behavioral types.

In summary, we assembled a unique data-set comprising ten different poeciliid species (in some cases, several sub-species or ecotypes, or multiple populations) and sought for variation at the taxon level (“species-specific behavioral types”) in (1) audience-induced changes in male mate choice, (2) deceptive male mating behavior, (3) sexual activity (previously published, re-analyzed own data, see Table 1), and (4) aggressiveness (newly generated data as well as previously published own data, Table 1). We tested for correlations of these behavioral tendencies, *i.e.*, we asked whether there are behavioral syndromes at the taxon level.

**Table 1.** The mean (± SE) standard length (SL [mm]) of the test fish used in the experiments examining ( *a*) male aggressiveness and ( *b*) male sexual behavior and mate choice. In ( *a*) SL differences between the two opponents are given along with the results from paired *t*-tests comparing winner and loser SL after dominance was established. In ( *b*) *N*_audience_ indicates the number of trials with an audience presented during the second part. * indicates species imported by “ *Aquarium Dietzenbach GmbH*”.

( *a*) Aggressive behavior	*N*_dyads_	Dyad SL	SL difference	*t*	*df*	*P*	Source
*G. sexradiata*	8	18.6 ± 0.7	1.3 ± 0.3	1.09	5	0.33	this study
*H. milleri*	14	22.5 ± 0.5	1.2 ± 0.2	1.41	3	0.25	this study
*P. reticulata* (feral)	8	16.2 ± 0.8	1.1 ± 0.2	0.58	3	0.60	this study
P. reticulata*	11	22.5 ± 0.7	1.2 ± 0.3	0.00	6	1.00	this study
P. picta*	9	23.3 ± 0.6	2.3 ± 0.4	0.36	6	0.73	this study
L. tridens*	18	24.8 ± 0.8	1.5 ± 0.2	0.37	7	0.72	this study
*L. sulphurophila*	12	32.7 ± 1.1	1.9 ± 0.4	2.11	11	0.58	this study
*P. latipinna*	9	43.6 ± 2.8	2.2 ± 0.4	0.01	5	1.00	this study
*P. latipunctata*	9	25.3 ± 1.3	1.4 ± 0.3	1.57	3	0.22	this study
*P. orri*	9	33.1 ± 0.7	2.0 ± 0.4	1.20	8	0.27	this study
*P. m. limantouri*	12	37.1 ± 1.8	2.7 ± 0.4	1.01	10	0.30	[ 51]
*P. m. mexicana* (sulfide)	9	28.7 ± 0.9	2.0 ± 0.4	2.05	8	0.12	[ 51]
*P. m. mexicana*	18	35.7 ± 1.3	1.4 ± 0.3	1.06	15	0.27	[ 51]
( *b*) Male mating behavior	*N*_trials_	Focal male SL	Large female SL	Small female SL	*N*_audience_	Audience male SL	Source
*G. sexradiata*	20	21.4 ± 0.6	37.9 ± 0.7	30.3 ± 0.6	10	21.7 ± 0.6	[ 42]
*H. milleri*	25	22.4 ± 0.5	33.3 ± 0.5	25.0 ± 0.7	14	21.4 ± 0.5	[ 58]
*P. reticulata* (feral)	32	14.9 ± 0.2	19.3 ± 0.4	14.8 ± 0.2	16	14.9 ± 0.2	[ 55]
P. reticulata*	47	21.8 ± 0.4	33.4 ± 1.1	24.2 ± 1.0	25	21.2 ± 0.5	[ 42]
P. picta*	43	23.0 ± 0.2	34.0 ± 0.8	26.7 ± 0.6	26	22.6 ± 0.4	[ 42]
L. tridens*	46	23.6 ± 0.3	30.2 ± 0.9	25.6 ± 0.2	23	22.6 ± 0.4	[ 42]
*L. sulphurophila*	28	31.0 ± 0.7	38.6 ± 1.0	31.6 ± 0.8	14	32.2 ± 0.8	[ 42]
*P. latipinna*	31	36.4 ± 1.0	45.4 ± 0.5	33.8 ± 0.8	18	35.2 ± 1.0	[ 42]
*P. latipunctata*	21	25.9 ± 0.8	35.0 ± 0.5	27.6 ± 0.5	11	25.5 ± 0.8	[ 42]
*P. orri*	18	32.2 ± 0.8	37.8 ± 0.8	32.1 ± 0.7	9	32.4 ± 1.0	[ 42]
*P. m. limantouri*	36	34.0 ± 0.9	49.9 ± 0.4	33.8 ± 0.6	18	35.8 ± 1.0	[ 41]
*P. m. mexicana* (sulfide)	22	29.0 ± 0.6	47.6 ± 1.3	35.3 ± 0.6	11	30.2 ± 0.7	[ 42]
*P. m. mexicana*	39	32.5 ± 1.0	47.4 ± 0.8	37.4 ± 0.8	19	35.2 ± 1.3	[ 42]

## Methods

### Study organisms and their maintenance

The experiments reported here comply with the current laws of Germany (approved by Regierungspräsidium Darmstadt V-54-19c-20/15-F104/Anz.18) and the USA (approved by the Institutional Animal Care and Use Committee of the University of Oklahoma; AUS-IACUC approved protocols: R06-026 and R09-023).

Test subjects were lab-reared descendants of wild-caught fish. We included Atlantic mollies from the coastal lagoons around the Central Mexican city of Tampico (belonging to the subspecies *P. mexicana limantouri*); another population was collected in the Río Oxolotan in Tabasco, South México ( *P. mexicana mexicana*). Recent phylogenetic analyses argue in favor of full species status of the two subspecies ^48^. We further included a locally adapted and genetically differentiated ( *i.e.*, independently evolving) ecotype from the *P. mexicana mexicana* clade: the hydrogen sulfide-adapted form inhabiting El Azufre, a tributary to the Río Oxolotan ^49, 50^. As another representative of short-fin mollies ^23, 51^ we included mangrove mollies ( *P. orri*) from Roatán Island, Honduras. Two species of long-fin mollies were tested: sailfin mollies ( *P. latipinna*) stemmed from the Comal River in Central Texas, USA, while Tamési mollies ( *P. latipunctata*) were collected near Ciudad Mante in Tamaulipas, México. We further included guppies ( *P. reticulata*) from Venezuela and a feral population from the San Antonio River, Texas, USA ^52^, as well as Venezuelan swamp guppies ( *P. picta*). As representatives of the genus *Limia*, we included *L. tridens* and sulfur limia ( *L. sulphurophilia*), both originating from the Dominican Republic. *Gambusia sexradiata* from the Río Teapa, and Grijalva mosquitofish ( *Heterophallus milleri*) from the Río Oxolotán (both Tabasco, México) were included as representatives of mosquito fishes.

Test fish came from large, randomly outbred single-species stocks maintained at the Department of Ecology and Evolution of the University of Frankfurt ( *P. m. mexicana*, *P. m. limantouri*, *P. reticulata* from Venezuela, *P. picta*, *L. tridens*), or at the Department of Zoology at the University of Oklahoma in Norman ( *P. m. mexicana* from El Azufre, *P. latipinna*, *P. latipunctata*, *P. orri*, feral *P. reticulata*, *L. sulphurophila*, *G. sexradiata, H. milleri*; Table 1). Fish were reared as single-species, mixed-sex stocks in 200-l (Frankfurt) or 1,000-l (Norman) tanks at 25–27°C under an 12:12 hours light: dark cycle (Frankfurt) or under ambient light conditions in a greenhouse (Norman). At the University of Frankfurt, fish were fed twice daily *ad libitum* with commercial flake food. Stock tanks in Norman contained naturally growing algae as well as a variety of naturally occurring invertebrates such as chironomid larvae, copepods and amphipods, on which the fish could feed. In addition, fish were supplied with flake food every two days. However, at least 1 week prior to the behavioral experiments, fish were fed *ad libitum* at least once daily with flake food.

## Experimental design

### Aggressive behavior

We determined male aggressive behaviors during dyadic encounters by analyzing contests staged between pairs of males in a small test tank measuring 30 × 20 × 20 cm ^53^. To avoid confounding effects of previously established dominance and/or familiarity ^54, 55^, males were taken from different stock tanks. Males in a dyad differed by less than 15% in standard length (SL), which has previously been established as the threshold below which fights typically escalate ^53^; nevertheless, size difference was included as a covariate in the statistical analyses (see below). We separated males by an opaque filter sponge while three sides of the test tank were taped with gray paper to minimize disturbances from the outside. The bottom of the tank was filled with black gravel, and water was aerated and maintained at 27–29°C. Males could habituate to the test tank overnight, and observations took place the next day between 09:00 and 13:00. To initiate a trial, the sponge divider was gently lifted, and we noted behavioral interactions for a maximum of 10 minutes, starting with the first interaction. We focused on three frequent aggressive behaviors ^56, 57^: (1) S-position: this threat display usually initiates a fight. Males swim in a parallel or anti-parallel position and bend their bodies in an S-shaped manner with all unpaired fins erect; (2) tail-beats: S-positions are often followed or superimposed by tail-beats, which are fast movements of head and tail in opposing directions that either touch the opponent’s body or send shock waves to the opponent; and (3) bites – we defined all incidences of ramming and bite-like attacks as bites, because both these behaviors occur extremely quickly and thus are indistinguishable to the human eye. For some species examined in this study no formal description of aggressive behavior was available from the literature, and so we confirmed in pre-trials that the aforementioned behaviors are part of their behavioral repertoire.

We also recorded fight duration until dominance was established. Contest outcome could be inferred from behavioral differences between the contestants. Folded fins, head-down posture and a position at the periphery of the tank typically characterize contest losers, while winners constantly chase and further attack the loser with fins fully erect, occasionally performing S-positions or bites ^53^. We met all requirements for animal well-being in behavioral experiments; apart from the occasional loss of single scales, no severe injuries were observed, as we separated males immediately once dominance was established. If no dominance was established within 10 minutes of the first interaction, we terminated the fight; those trials were discarded from the analysis of fighting durations ( *N* = 52 cases discarded), while fight durations were scored as “0” when no aggressive behavior occurred at all (those trials were terminated after a total of 15 minutes of observation). SL of both contestants was taken after a contest by laying the fish flat on plastic foil-covered millimeter paper ( Table 1). Afterwards we transferred males back to their respective stock tanks. In total, we successfully completed *N* = 146 trials ( Table 1).

### Male mate choice

We reanalyzed previously published data on audience-induced changes in male mate choice ( Table 1). Focal males were isolated in 25- to 38-l tanks for two to four days prior to the tests to ensure that they were motivated to mate ^12^. We tested each focal male only once; however, owing to the limited number of males available from our stocks, some males were also used as audience males after they had served as a focal male, but never on the same day and not in the same dyadic constellation. As familiarity among males affects the strength of audience effects in *P. mexicana* ^9^, focal and audience males were taken from different stock tanks.

Each focal male was tested for its mating preference in a binary choice situation and was then retested with the same stimulus females either without audience (control treatment) or with an audience male present (50% of trials each). We were thus able to examine changes in focal males’ behavior from the first to the second part of the tests and could discern between effects induced by the audience and changes that would occur over the course of the experiment even without audience. In theory, we could have used an alternative design of presenting an audience in all trials while starting the tests with or without audience in alternating order; however, in such a design, prior exposure to the audience male (when presented during the first part) could still affect the focal males’ behavior during the second part of the tests ^58^.

The test tank (50 × 30 × 30 cm, length × width × height) was filled to 20 cm height with aged tap water. Water temperature was maintained at 27–28°C using an aquarium heater. In addition, the water was aerated between trials, but both the heater and the air-stone were removed for all trials. Black plastic covered all sides except the front. Prior to the tests, we choose two different-sized stimulus females (for SL see Table 1) from a stock tank and introduced them into the test tank. Poeciliid males prefer to mate with larger, more fecund females ( *e.g.*, ^8, 59– 61^, but see Baerends *et al.* ^62^). Afterwards, we introduced a focal male into a transparent Plexiglas cylinder (10 cm diameter) located in the center of the tank and left the fish undisturbed for 5 minutes. After the habituation period, we gently lifted the cylinder. During a 10-min observation period, we scored male sexual behaviors directed toward either of the two females and noted with which female the focal male interacted with first. We decided *a priori* to terminate trials if the male did not show any sexual behavior during the first part of the test; *N* = 3 trials with *P. orri*, *N* = 5 ( *P. latipinna*), *N* = 2 ( *P. latipunctata*), *N* = 4 ( *P. reticulata*, Venezuela), *N* = 1 ( *P. picta*), *N* = 1 ( *P. reticulata*, San Antonio), and *N* = 6 ( *H. milleri*) were discarded from the statistical analyses based on this criterion.

Genital nipping is a typical pre-copulatory behavior in poeciliids, whereby the male approaches the female from behind and touches her genital region with his snout ^30, 56^. During thrusting, males swing their gonopodium forward while attempting to introduce it into the female’s gonopore. However, in most poeciliids it is not possible to discriminate with certainty between a successful mating (defined as a mating with sperm being transferred) and the pure mating attempt. Courtship behavior is absent in *P. mexicana* ^30^, *P. orri*, the examined *Limia* species (authors, personal observation) and *Gambusia* spp. ( ^63^ for *G. holbrooki*). *Poecilia reticulata* males court in front of females in an S-shaped body posture (sigmoid displays ^64, 65^), while the primary courtship display of *P. picta* males consists of circling around the female (the so-called ‘orbit’ ^56, 65^), but males also court with their fins raised in front of the female ( ^65^; D.B., personal observation). *Heterophallus milleri* males circle around the female and swing their gonopodium forward when in the female’s visual field ^61^. Large *P. latipinna* and *P. latipunctata* males occasionally court in front of females with raised dorsal fins ^56, 66^. As not all species examined herein show courtship displays and courtship was by far the least frequent behavioral category, we excluded numbers of courtship displays from our main analyses.

Upon completion of the first preference test, we immediately repeated measurement of male mating preferences, but in one half of the trials, an audience male was presented, while the other half of the trials was repeated without audience (control). To initiate this second part of a trial, we reintroduced the focal male into the acclimatization cylinder. An audience male was placed in another transparent cylinder in the central back of the tank, while for the control only an empty cylinder was presented. The audience male was confined in his cylinder throughout the test. After another 5 minutes of habituation (during which all four fish could interact visually), measurement of male preferences was repeated, as described above. Interactions between males were not quantified, but aggressive displays were not observed. In total, we successfully completed *N* = 408 trials ( Table 1). Once a trial was completed, all fish were measured for SL to the closest millimeter ( Table 1).

## Statistical analyses

First, we asked whether species show consistent variation in the behavioral traits examined in this study (on the individual level often referred to as “character” or “behavioral type”, *e.g.*, ^42^). In analogy to individual-level analyses of behavioral consistency (where each individual is tested repeatedly), our species-level analysis defined each tested individual as a repeated measure of the subject ‘species’. We used univariate mixed models (MM) in which we treated the mean of each behavioral trait as a fixed effect and included random intercepts for each species. This approach was recently recommended to decompose phenotypic variance into a within-subjects variance component ( *i.e.*, the variance around the species-specific intercept) and a between-subjects variance component ( *i.e.*, the variance between species-specific intercepts) ^67^. Consistent differences among species – species-specific ‘behavioral types’ – for a given behavioral trait can be inferred when the between-subjects variance component significantly differs from zero. Based on the variance decomposition through MMs, we furthermore calculated a metric for the repeatability of each behavioral trait, *i.e.*, the proportion of the total variance accounted for by differences among species ( *sensu* ^68^):

$$R=\frac{Variance\;(between\;species)}{Variance\;(between\;species)\;+\;Variance\;(within\;species)}$$

The three members of the *Poecilia mexicana* species-complex used in our study clearly represent three phylogenetically independent groups (two sub-species and one derived ecotype ^48^) and, thus, were treated statistically as independent species. However, this was not the case for the two populations of the guppy ( *P. reticulata*) and so we re-ran all analyses without data from the feral guppy population (San Antonio), but this did not alter the direction of the results (not shown).

We then proceeded to ask whether the different behavioral traits are correlated among species ( *i.e.*, if behavioral syndromes can be inferred; ^39^). To this end, we calculated pair-wise non-parametric Spearman’s rank correlations with species means for all behavioral traits. We are aware of other methods to test for a syndrome structure, namely, multivariate MMs ^67^, but based on our limited sample size of *N* = 13 independent subjects (species/populations) we decided to use non-parametric tests instead (which is also an accepted technique, see ^69^).

We depict mean values (± standard error) of the investigated behaviors for all species examined.

### Aggressive behavior

In order to compare variation in aggressive behavior across species, we employed Principal Component Analysis (PCA) to reduce the number of dependent variables (numbers of S-positions, tail-beats and bites per male dyad) and extracted one independent component (PC1; eigenvalue = 2.47) that explained 82.3% of the variance. The three aggressive behaviors had axis loadings of 0.85 (S-positions), 0.93 (tail-beats) and 0.94 (bites). PC1 was checked for normal distribution using a Kolmogorov-Smirnov test and used as dependent variable in a linear mixed model (LMM, ‘mixed’ procedure in SPSS 21) with species-specific random intercepts (see above). To test whether the variance between intercepts differed significantly from zero (thus indicating consistent differences between species in aggressive behavior) we compared a model with random intercepts to a reduced model without random intercepts via likelihood ratio tests. Male body size may influence aggressiveness ^70^, and this could affect apparent between-species effects (with larger species being more aggressive than smaller ones) as well as within-species effects (larger males within a given species can be more aggressive than smaller ones). However, the within-species effect of body size can also vary between species (when larger males are more aggressive than smaller ones in one species but not in another). To separate within- from between-species effects, we followed the “within-subject centering” approach proposed by van de Pol and Wright ^71^ and included species means for the mean SL of a dyad (termed ‘between-species dyad SL’) as well as each dyad’s deviation from the respective species mean (termed ‘within-species dyad SL’) as fixed covariates in our model. To test whether the within-species effect of mean dyad SL differed between species, we included random slopes of ‘within-species dyad SL’ for each species in our model and tested for slope heterogeneity through likelihood ratio tests (model with random slopes vs. model without random slopes, see ^67^). Furthermore, the opponents’ body size difference influences fight intensity ^53^, which again can be a species-specific trait. As our experimental setup largely prevented between-species variation in ‘opponent body size difference’ as we had chosen pairs of males that differed by less than 15% in SL, we were interested in whether fights with smaller SL differences between both opponents were more intense than fights with larger differences and thus included ‘opponent body size difference’ (arcsine (square root)-transformed SL _small_/SL _large_) as a fixed covariate. To test whether there were between-species differences in the effect of ‘opponent body size difference’ we again included species-specific random slopes and tested for slope heterogeneity using likelihood ratio tests. Non-significant fixed effects and random slopes were excluded from the final model. We, thus, excluded the covariates ‘between-species dyad SL’ (estimated slope: 0.013 ± 0.021; *F*_1,11.24_=0.37; *P*=0.54), ‘within-species dyad SL’ (estimated slope: -0.039 ± 0.190; *F*_1,119.9_=4.00; *P*=0.12) and random slopes for ‘within-species dyad SL’ (estimated variance: 0.003 ± 0.003; *P*=0.12) from the final model.

Repeatability was calculated based on the final model; as we retained the covariate ‘opponent body size difference’ as a fixed effect with random slopes for each species in the model, our measure of repeatability represented conditional repeatability ^67^ where opponent body size difference equals zero.

Fight durations (log-transformed prior to the analysis to approach normal error distribution) were analyzed in a similar LMM. All three covariates and random slopes were removed from the final model since none was significant (‘between-species dyad SL’, estimated slope: -0.017 ± 0.021; *F*_1,11.1_=0.66, *P*=0.434; ‘within-species dyad SL’, estimated slope: -0.027 ± 0.031; *F*_1,2.1_=0.76, *P*=0.47; random slopes for ‘within-species dyad SL’, estimated variance: 0.003 ± 0.007; *P*=0.58; ‘opponent body size difference’, estimated slope: -1.435 ± 0.897; *F*_1,61.3_=2.55, *P*=0.11; random slopes for ‘opponent body size difference’, estimated variance: 0.408 ± 2.241; *P*=0.84). Repeatability was calculated as described before.

### Male sexual behavior

As a measure of sexual activity we used numbers of sexual behaviors directed to both stimulus females in the first part of a mate choice trial (without audience male). As described for the analysis of aggressive behavior, we used PCA to condense sexual behavior (genital nipping and thrusting) to one principle component (PC1, eigenvalue = 1.79) that explained 89.7% of the total variance. Both variables had equal axis loadings of 0.95. We used PC1 (checked for normal distribution by means of a Kolmogorov-Smirnov test) as dependent variable in a LMM (see above). Small males show more sexual behaviors than larger ones in at least some of the species examined here as part of a ‘sneak-like’ alternative mating strategy ^72^, so we included species-wise means for focal males’ SL (‘between-species focal SL’) as well as each focal male’s SL deviation from the species mean (‘within-species focal SL’) as fixed covariates. As described for aggressive behavior, we included species-specific random slopes for the within-species covariate to test for between-species differences in the relation between sexual activity and focal males’ body size. Also, poeciliid males typically prefer to mate with large females, and so we included the SL difference of each stimulus female dyad [arcsine (square root)-transformed SL _small_/SL _large_] as another fixed effect covariate and accounted for potential between-species differences by including random slopes. However, all three covariates and the random slopes had no significant effect (‘between-species focal SL’, estimated slope: 0.049 ± 0.035; *F*_1,10.9_=1.94, *P*=0.19; ‘within-species focal SL’, estimated slope: 0.017 ± 0.010; *F*_1,393.9_=2.94, *P*=0.087; random slopes for ‘within-species focal SL’, estimated variance: 0.002 ± 0.010; *P*=0.67; ‘stimulus SL difference’, estimated slope: 0.083 ± 0.380; *F*_1,398.1_=0.05, *P*=0.83; random slopes for ‘stimulus SL difference’, estimated variance: 0.002 ± 0.007; *P*=0.72), and were removed from the final model. Repeatability was calculated based on the final model as described for aggressiveness.

### Audience-induced changes in preference expression

To compare the magnitude of audience-induced changes in individual male mate choice behavior across species, we calculated a preference score ^36^ as:

(fraction of sexual behaviors with the initially preferred female during the second part of a trial) – (fraction of sexual behaviors with the same female during the first part),

such that negative values would indicate that individual preferences decreased. We analyzed scores as dependent variable in a LMM with species-specific random intercepts and ‘treatment’ as another random factor. ‘Treatment’ was also used as a fixed factor such that we could evaluate first whether there was an overall treatment effect on the dependent variable and secondly decompose the variance into treatment-specific between- and within-species components. Again, focal male body size as well as stimulus size difference could have influenced preference expression and so we initially included ‘between-species focal SL’, ‘within-species focal SL’ and ‘stimulus SL difference’ as fixed covariates (and random slopes for the latter two) but removed them from the final model as none had a significant effect (‘between-species focal SL’, estimated slope: 0.004 ± 0.010, *F*_1,10.9_=1.62, *P*=0.22; ‘within-species focal SL’, estimated slope: -0.001 ± 0.004, *F*_1,347.3_=0.16, *P*=0.68; random slopes for ‘within-species focal SL’, estimated variance: -0.007 ± 0.012, *P*=0.27; ‘stimulus SL difference’, estimated slope: 0.136 ± 0.130, *F*_1,308.1_=1.09, *P*=0.30; random slopes for ‘stimulus SL difference’, estimated variance: -0.003 ± 0.004, *P*=0.41). Repeatability was calculated for both treatments separately, as described above.

### Deceptive mating behavior

The first sexual approach of focal males is assumed to be another indicator of male preference ^36^. We sought to corroborate this assertion and thus, tested whether males on average interacted more with the females they approached first in the first part of our tests. In all species most males first approached the female they also interacted most often with during the entire first preference test (in 76–100% of trials those females approached first also received the majority of sexual behaviors; *chi* ^2^-tests significant for all species, results not shown). In the context of deceptive mating behavior, the first sexual approach of focal males is of interest as interacting first with the previously non-preferred female has been interpreted as an attempt to mislead the rival ^36^. Thus, we analyzed the fraction of males that first interacted with the opposite (“1”) or same female during the second part (“0”) using a Generalized Linear Mixed Model (GLMM) with a binary error distribution and a logit-link function. As described for the LMMs analyzing audience-induced changes in mating preferences, ‘species ID’ was used as a grouping variable in combination with ‘treatment’, while ‘treatment’ also served as a fixed factor. We initially included ‘between-species focal SL’, ‘within-species focal SL’ and ‘stimulus SL difference’ as fixed covariates but removed them from the final model as they had no significant effects (‘between-species focal SL, estimated slope: -0.005 ± 0.022, *F*_1,398_=0.052, *P*=0.82; within-species focal SL’, estimated slope: 0.078 ± 0.560, *F*_1,398_=0.044, *P*=0.89; ‘stimulus SL difference’, estimated slope: 0.888 ± 1.157; *F*_1,399_=1.53, *P*=0.22). It was not possible to fit random slopes in the GLMM model, but as neither covariate had a significant effect, differences between species likely can be neglected. Repeatability was calculated for each treatment separately based on the variances obtained from the final model, and thus represents link-scale repeatability ^69^.

### Correlations of behavioral types at the species level

The central question of our present paper was whether there are correlations between the aforementioned behaviors at the species level. Owing to the limited sample size ( *N* = 13 groups), we used non-parametric, pair-wise Spearman’s rank order tests to correlate species means for (1) aggressiveness (log(sum of aggressive interactions per fight)), (2) fight duration (log(time)), (3) sexual activity (sum of nipping and thrusting behavior during the first part of the tests), (4) consistency in preference expression without an audience (preference score), (5) the strength of changes in preference expression when an audience male was presented (preference score), (6) consistency in first approached females without an audience male presented (fraction of males that changed their first interaction without audience present), (7) deceptive male mating behavior (fraction of males that changed their first interaction in the audience treatment). We are aware of a possible error inflation due to multiple comparisons, but did not use alpha-corrections (such as Bonferroni) since the investigated behaviors were not independent. To further show the intercorrelative character of the investigated behaviors, we condensed them through PCA and extracted two principle components with Eigenvalues above 1 (Eigenvalues: PC1=2.49; PC2=1.92) that explained 35.5% and 27.6% of the total variation, respectively. The principle components were varimax-rotated for better interpretation.

## Results

### Male aggressive behavior

**Figure 1.**
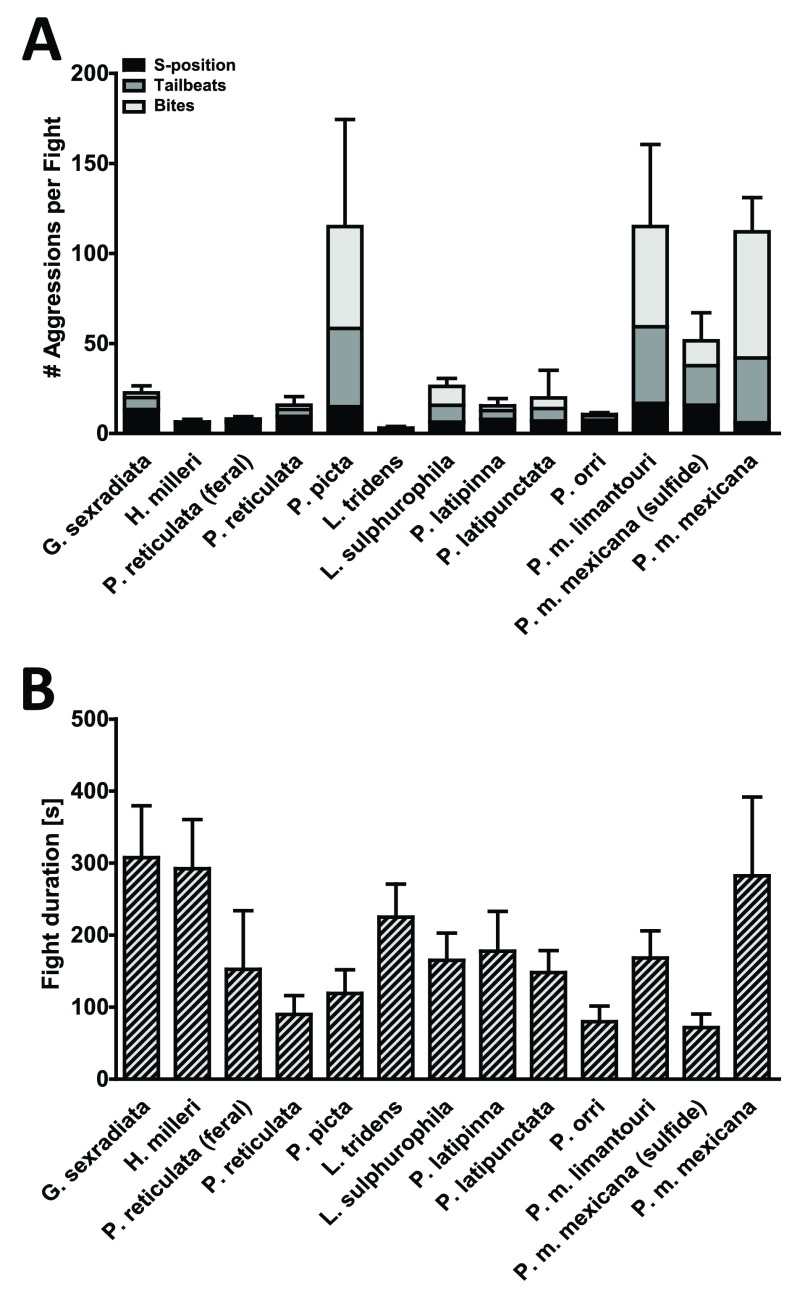
Means (± SE) of ( **a**) numbers of aggressive interactions per male fight and ( **b**) fight duration in the different poeciliid species examined.

There was significant between-species variation in aggressiveness ( Table 2a) indicating that some species are consistently more aggressive than others ( Figure 1a). On average, the amount of aggressive behaviors decreased with increasing size-difference between the opponents even though this effect was not significant when random slopes for each species were included (fixed covariate ‘opponent body size difference’: estimated slope: -1.492 ± 1.249, *F*_1,12.9_=1.60, *P*=0.23). Nevertheless, species-specific random slopes differed significantly between species (variance estimate: 13.020 ± 6.923; *P*<0.001) and were negatively correlated with the species-specific random intercepts ( *r*_intercept-slope_=-0.95, *P*<0.001) indicating that highly aggressive species reduced aggressive behavior more when opponent SL difference increased than less aggressive species. The repeatability value — by inclusion of random slopes for opponents’ body size difference representing the conditional between-species variance at an extrapolated opponent body size difference of zero — was relatively high at 0.71 ( Table 2a).

**Table 2.** Results from mixed models (LMM, linear mixed model; GLMM, generalized linear mixed model) analyzing. ( *a*) aggression in dyadic male fights, ( *b*) male sexual behavior in dichotomous choice tests, ( *c*) changes in male preference expression with or without an audience male presented, and ( *d*) deceptive male mating behavior with or without an audience male present. The between- ( *Var*_between_) and within-species variances ( *Var*_within_), and the associated repeatability values ( *R*) are shown. Significant between-species variances ( *P*-values obtained from likelihood ratio tests) are given in bold typeface.

Test	Dependent	*Var*_between_	*P*	*Var*_within_	*R*
*(a) Aggressive behaviors (LMM)*					
Number of aggressive behaviors	**PC1**	**1.38**	**< 0.001**	**0.57**	**0.71**
Fight duration	**ln(duration)**	**0.19**	**0.008**	**0.74**	**0.20**
*(b) Sexual activity (LMM)*					
Number of sexual behaviors 1 ^st^ part	**PC1**	**0.64**	**< 0.001**	**0.51**	**0.55**
*(c) Change in mating preference (LMM)*					
Without audience	Preference score	0.003	0.24	0.055	0.048
With audience	Preference score	0.006	0.19	0.085	0.065
*(d) Deception (GLMM)*					
Without audience	Number of males that changed 1 ^st^ interaction	0.21	0.10	0.960	0.18
With audience	**Number of males that** **changed 1 ^st^ interaction**	**0.58**	**0.002**	**0.95**	**0.38**

When analyzing fight durations, we again found significant variation between species ( Table 2a, Figure 1b), while repeatability was much lower than for numbers of aggressive behavior ( Table 2a).

### Male sexual behavior

**Figure 2.**
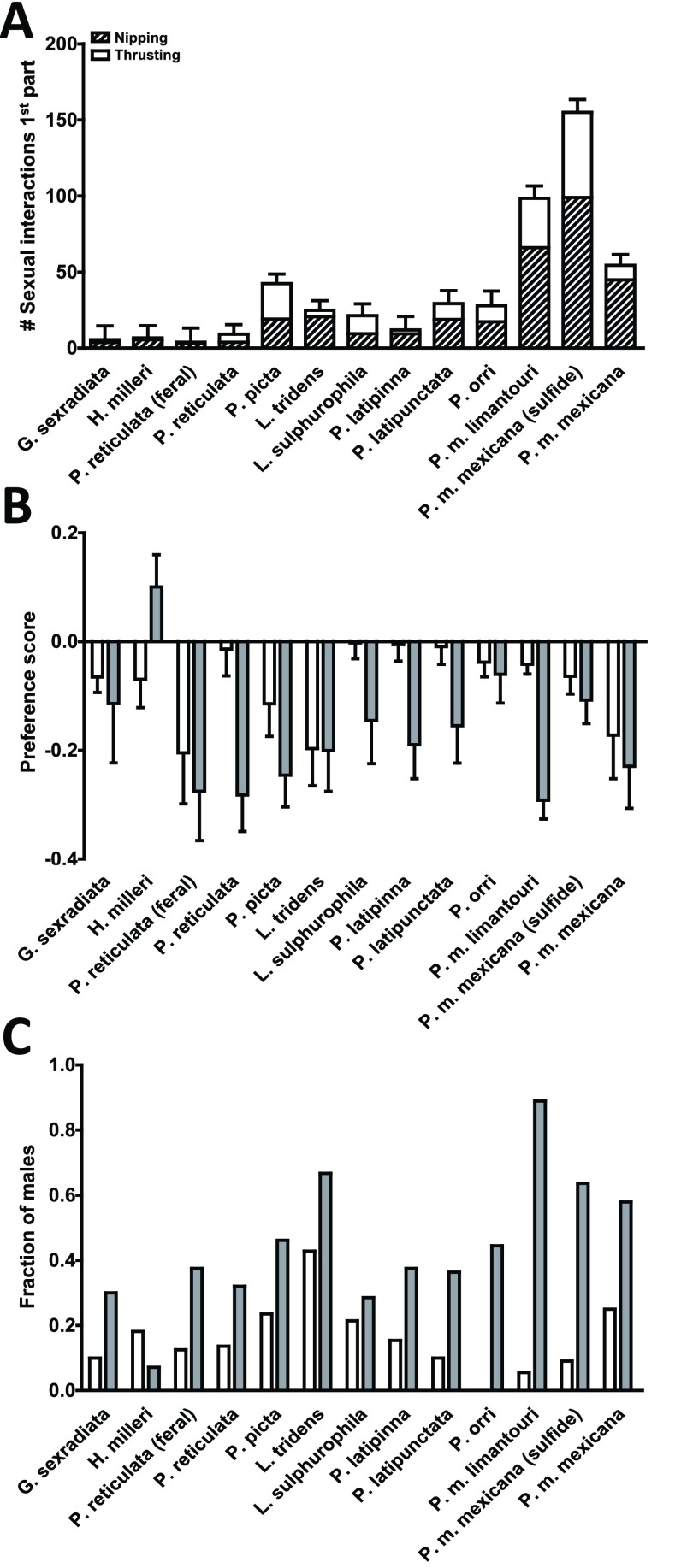
( **a**) Mean (± SE) numbers of male sexual behaviors during the 10 min observation period. ( **b**) Changes in individual focal males’ mate choice behavior in the presence of an audience male. Depicted are mean (± SE) preference scores (see main text), whereby negative values indicate that male preferences decreased in strength. ( **c**) Proportion of males that first interacted with the opposite female when released from the acclimation cylinder in the second part of the tests. Open bars in ( **b**) and ( **c**) represent the control treatment (no audience) while gray bars represent the audience treatment.

There was pronounced variation among species in male sexual activity ( Table 2c) with some species (especially Atlantic mollies) being far more active than others ( Figure 2a). Repeatability for sexual activity was comparably high as for aggressive behavior ( Table 2b).

### Audience-induced changes in preference expression

When comparing the change in individual males’ mating preferences from the first to second part of the tests (preference score), we detected no significant between-species variation — both with and without an audience male presented ( Table 2c). The fixed factor ‘treatment’ had a significant effect ( *F*_1,21.5_=6.87, *P*=0.016) indicating that preference scores, overall, differed in response to whether or not an audience male was presented. All species except *H. milleri* showed similar responses: males were consistent in their mate choice behavior when no audience male was presented and decreasing preferences when observed by an audience ( Figure 2b).

### Deceptive mating behavior

Our GLMM did not detect significant between-species variation in fractions of males that changed the initially preferred female from the first to the second part of the mate choice tests when no audience was presented ( Table 2d). In other words: species were similarly consistent in their preferences in the control treatment. In the treatment where an audience male was presented during the second part of the test, we found significant between-species variance, along with a comparably high repeatability value ( Table 2d). The fixed factor ‘treatment’ was significant in our final model, indicating that males were generally more likely to interact with the opposite species in the treatment involving an audience male ( Figure 2c).

### Correlations of behavioral types at the species level

**Figure 3.**
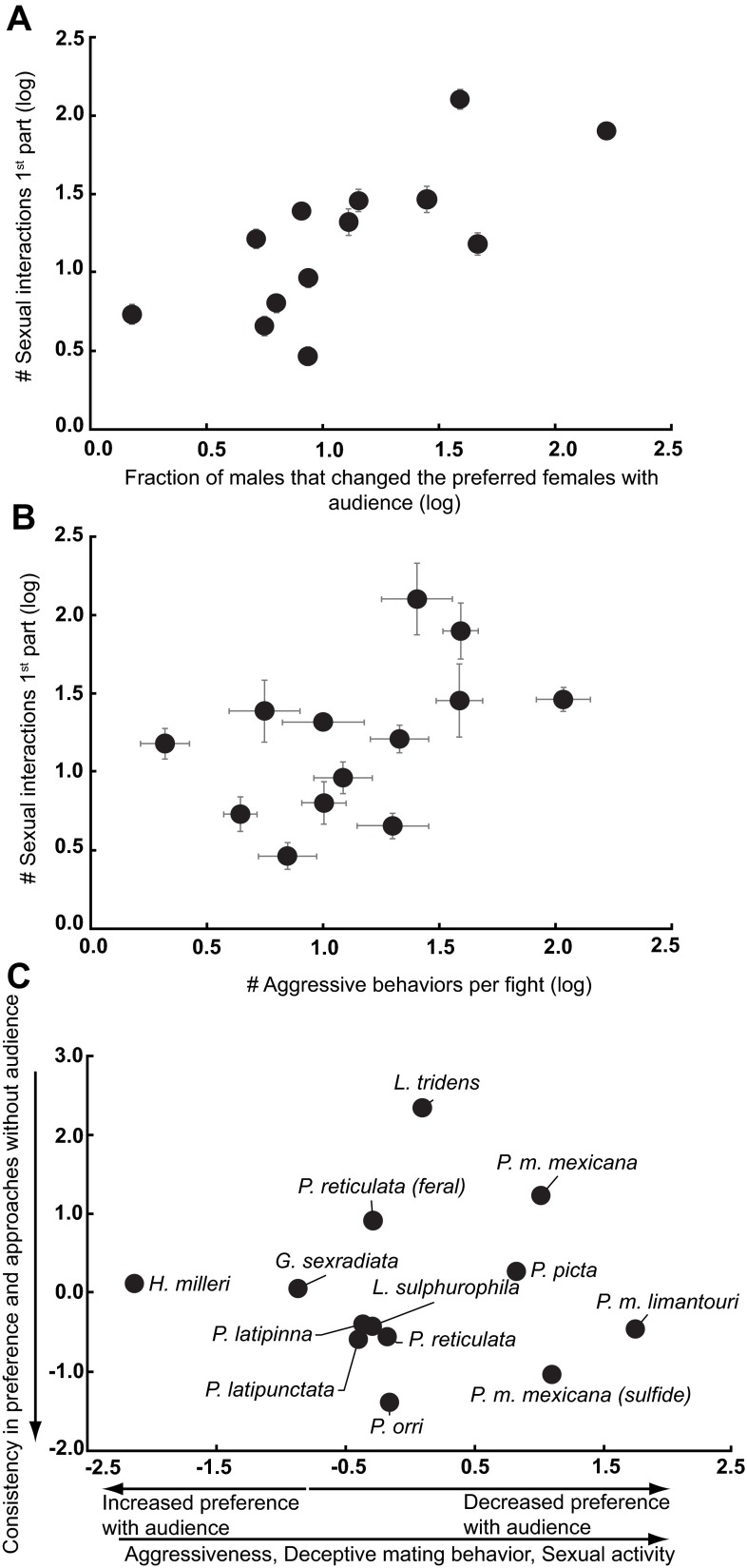
Correlations between species-level means (± SE) of male aggressiveness (log(number of aggressions per fight)) and ( **a**) sexual activity (log(number of sexual interactions during the 1 ^st^ part)) and ( **b**) deceptive mating behavior (fraction of males that changed the first approached female from 1 ^st^ part to 2 ^nd^ test part with an audience present). ( **c**) First two principle components from PCA with species-level means of all seven behaviors determined in this study. Axis loadings >0.63 are given along the respective axes.

In line with our prediction derived from the interpretation that SCR explains the occurrence of audience-induced behavioral changes, we found a strong, positive correlation between sexual activity and the amount of deceptive behavior at the species level ( Figure 3a). The alternative prediction, that avoidance of aggressive behavior drives audience effects (leading to positive correlations between the degree of preference change and aggressiveness as well as between deceptive behavior and aggressiveness), received no support (not statistically significant; Table 3). However, there was also a significant positive correlation between the amount of aggressive behavior and sexual activity ( Figure 3b).

**Table 3.** Pair-wise Spearman’s rank order correlations between species-wise mean values for all seven behaviors investigated. An asterisk indicates significant correlations.

Behavior	Aggressiveness	Fight duration	Change in preference (without audience)	Change in preference (with audience)	Sexual activity	Change in first interaction (without audience)	Change in first interaction (with audience)
**Aggressiveness**	-	*r*_s_=-0.21; *P*=0.48	*r*_s_=0.07; *P*=0.82	*r*_s_=-0.31; *P*=0.31	*r*_s_=0.61; *P*=0.027*	*r*_s_=-0.05; *P*=0.88	*r*_s_=0.36; *P*=0.23
**Fight duration**		-	*r*_s_=-0.22; *P*=0.47	*r*_s_=0.15; *P*=0.62	*r*_s_=-0.33; *P*=0.27	*r*_s_=0.41; *P*=0.16	*r*_s_=-0.28; *P*=0.36
**Change in preference** **(without audience)**			-	*r*_s_=0.19; *P*=0.54	*r*_s_=0.13; *P*=0.68	*r*_s_=-0.35; *P*=0.24	*r*_s_=-0.34; *P*=0.26
**Change in preference** **(with audience)**				-	*r*_s_=-0.08; *P*=0.79	*r*_s_=-0.18; *P*=0.57	*r*_s_=-0.41; *P*=0.16
**Sexual activity**					-	*r*_s_=-0.14; *P*=0.65	*r*_s_=0.67; *P*=0.013*
**Change in first interaction** **(without audience)**						-	*r*_s_=-0.06; *P*=0.84
**Change in first interaction** **(with audience)**							-

PCA with all seven behaviors retrieved two principle components accounting for 63.1% of the total variance. While PC1 received strongest loadings from deceptive male mating behavior (fraction of males that changed their first interaction in the audience treatment; axis loading: 0.90), sexual activity (0.78), aggressiveness (0.63) and preference changes due to an audience (-0.67; all other axis loadings between -0.40 and -0.01), PC2 received strongest loadings from both control treatments (change in preference without audience: -0.84; fraction of males that changed the initially approached female without audience: 0.85; all other axis loadings between -0.28 and 0.59) and thus reflects general consistency in mate choice behavior ( Figure 3c).

Data on dyadic male contests Data represents male aggressive behaviors during contests staged between pairs of males in a small test tank measuring 30 × 20 × 20 cm. We focused on three frequent aggressive behaviors: (1) S-position: this threat display usually initiates a fight. Males swim in a parallel or anti-parallel position and bend their bodies in an S-shaped manner with all unpaired fins erect; (2) tail-beats: S-positions are often followed or superimposed by tail-beats, which are fast movements of head and tail in opposing directions that either touch the opponent’s body or send shock waves to the opponent; and (3) bites – we defined all incidences of ramming and bite-like attacks as bites, because both these behaviors occur extremely quickly and thus are indistinguishable to the human eye. We also recorded fight duration until dominance was established. If no dominance was established within 10 minutes of the first interaction, we terminated the fight; those trials were discarded from the analysis of fighting durations, while fight durations were scored as “0” when no aggressive behavior occurred at all (those trials were terminated after a total of 15 minutes of observation). SL of both contestants was taken after a contest by laying the fish flat on plastic foil-covered millimeter paper.

## Discussion

Our current study identified aggressiveness, male sexual activity, and deceptive mating behavior in presence of an audience as consistent, species-specific behavioral traits, while decreased preference expression due to an audience (‘audience effects’ *sensu* ^37^) was found to be a universal feature in all but one of the investigated species. Also, species did not differ in their consistency during mate choice in the control treatment without audience — whether evaluated as the change in preference expression or numbers of males that changed the female with which they interacted first. Subsequent correlation analyses uncovered two effects: ( *a*) males of species with high sexual activity are more likely to show deceptive mating behavior, *i.e.*, they initially approached more often the non-preferred female when an audience male was presented; while species-level mean aggressiveness did not predict the occurrence of audience effects. ( *b*) Mean aggressiveness, by contrast, correlated positively with mean sexual activity. Hence, we detected two correlations of behavioral types at the species level.

One of the behavioral syndromes at the species level we uncovered in our present study — the correlation between aggressiveness and sexual activity — can be partly explained mechanistically through species differences in plasma concentrations of sexual corticosteroids (testosterone and its derivates ^73, 74^). Individual androgen concentrations predict aggressiveness in male swordtails, *Xiphophorus hellerii* ^75^; furthermore, plasma testosterone levels correlate positively with sexual behavior in male mosquito fish ( *G. holbrooki*) ^76^, so physiological pleiotropy could also explain species differences in aggression and sexual activity as detected here.

The main focus of our present study was on audience-induced changes in male mating behavior, and we asked if those behaviors can be linked to mean sexual activity and SCR. The rationale behind our prediction was that males of taxa with high overall sexual activity face a higher risk of by-standers making use of socially acquired information when eavesdropping on sexual interactions. It seems reasonable to assume the propensity for male mate choice copying to be a common feature of poeciliid mating systems ^10, 77^, but the likelihood of mate copying in natural systems should correlate positively with mean sexual activity. We found sexual activity (but not aggressiveness — despite some degree of inter-correlation between aggressiveness and sexual activity, see above) to correlate positively with the level of presumed deceptive mating behavior. This finding lends support to our hypothesis that SCR is a driving force behind the evolution of this behavior and is in line with our interpretation that focal males thus attempt to lead the rival away from their preferred mate, exploiting male mate choice copying to reduce SCR ^19, 21, 32^.

A general objection to our interpretation of deceptive mating behavior could be that leading the audience away from a preferred mating partner to deceive the rival may increase the risk of losing the preferred female, as poeciliid females tend to flee from male sexual harassment ^30, 78, 79^. We argue that this male behavior still offers advantages even if the preferred female flees: on the one hand, a pattern of last male sperm precedence was uncovered in guppies ^22, 80^, which renders mate choice copying a profitable option for the eavesdropping (copying) male ^10^. However, the longer the time between copulations by the first and second male in the mating trials conducted by Evans and Magurran ^80^, the higher the proportion of offspring fathered by the first male was. This implies that leading the by-standing rival away from (or at least delaying its approaches toward) a recently inseminated female would indeed be beneficial for the deceiving male even though it risks losing contact with the initially preferred (but already inseminated) female. Our interpretation assumes that males initially transferred sperm to the preferred female, which could not be determined unambiguously by simply counting copulation attempts. We thus recommend future experiments that will extract and quantify the amount of transferred sperm from females after the first preference test (see Evans *et al*. ^81^ for a protocol).

Since our analyses were based on species/population differences in aggressiveness, sexual activity and audience-induced changes in male mate choice behavior, we strongly recommend future experiments concentrating on within-population variation ( *e.g.*, individual “behavioral types”, ^42, 44^) that define a male’s response to a by-standing rival. For example, males are sensitive to the perceived sexual activity of a rival when exhibiting audience effects ^9^, and future studies could elaborate on the question of whether also perceived aggressiveness — a correlate of sexual activity — might influence the occurrence of audience effects. Such an experiment could also shed new light on the observed cross-correlation between sexual activity and aggressiveness as well as between sexual activity and deceptive behavior. However, such an approach requires multiple testing of the same individuals, which imposes logistic constraints on comparative analyses like our present study. Furthermore, future studies ought to elaborate on potential factors affecting the observed consistent behavioral differences among species. In this context, both phylogenetic considerations (for example through phylogenetically adjusted generalized linear models on a larger set of poeciliid species) and a comparison of shared and unique ecological features of different poeciliids are promising fields of investigation.

In summary, using a comparative approach, we were first able to quantitatively characterize behavioral types at the species level for several poeciliid species and further found correlational support for the hypothesis that SCR arising from male mate choice copying drives the evolution of audience-induced changes in male mate choice behavior. We argue that taxa with elevated sexual activity face a higher risk of males making use of socially acquired information ( *i.e.*, copying mate choice decisions), and so focal males in those species are more likely to respond to the presence of an audience with altered mate choice behavior.
