# Supplementary material for: Casanovas are liars: behavioral syndromes, sperm competition risk, and the evolution of deceptive male mating behavior in live-bearing fishes
Source: F1000Res. 2013 Oct 23;2:75. Originally published 2013 Mar 5. [Version 3] doi: 10.12688/f1000research.2-75.v3 (PMC3917653; doi:10.12688/f1000research.2-75.v3)
Supplement: Peer review history

# Peer review: Referee response for version 3

**Published:** 2013-11-14

**Authors:** Lisa Locatello

**Affiliations:** 1 Evolution and Ecology of Fish Reproduction, Department of Biology, University of Padova, Padova, Italy

In this updated version of the manuscript the Authors dealt with most of the raised issues.

I have read this submission. I believe that I have an appropriate level of expertise to confirm that it is of an acceptable scientific standard.

# Peer review: Referee response for version 2

**Published:** 2013-09-06

**Authors:** Lisa Locatello

**Affiliations:** 1 Evolution and Ecology of Fish Reproduction, Department of Biology, University of Padova, Padova, Italy

The Authors fulfilled properly all the statistical problems of the previous version and the manuscript is definitively improved.

However, even if the new analyses confirm the previous results, I still feel that the Authors’ conclusions are too far stretched and I would suggest a more cautious approach to the discussion. This is due to three main reasons:

1. The rationale behind predictions is that a higher sexual activity is linked with a higher SCR, but this is not directly demonstrated by this or by previous studies on the selected species. Therefore, I would be more cautious in definitively concluding that the positive correlation between sexual activity and deceptive mating behaviour support the hypothesis that SCR is the driving force behind the evolution of deception.

1. The firm conclusion that deceptive behaviour is independent from aggressiveness (derived from the absence of correlation between the two) has a week point if considering that sexual activity correlates with deceptive behaviour but it also correlates with aggressiveness. As a consequence the Authors’ should be less categorical in attributing the whole weight to sexual activity while totally excluding an implication of aggressiveness.

1. Main conclusions are drawn in the perspective of males that, after having inseminated a female, take advantage of a deceptive behaviour by standing rivals away from recently inseminated female even considering the risk of losing contact. But in fact, during the experiment it was impossible to discriminate a successful mating from a simple mating attempt. As a consequence, insemination (mating) should not be taken for granted. I would be more cautious in interpreting the adaptive behaviour of these experimental males as they have inseminated the female when this did not happen with certainty. Therefore, the Authors’ might want to consider the following assumptions with more prudence:

- i) That patterns of sperm precedence are the cause that renders the risk of losing a high quality and inseminated female beneficial. This may be true only if insemination definitely occurs.

-  ii) That male mate choice copying renders recently mated females more attractive to rivals, as they can’t be sure that experimental females have mated.

I have also a last minor comment on the phylogenetic comparative analyses that I suggested as a future perspective. By excluding the population of guppies that were most closely related to the Venezuelan guppies the Authors are not definitively controlling for independence of the results from phylogenetic relationship across species. 

What I meant is that only a phylogenetic comparative study, implying statistical analyses that account for phylogeny (see for example phylogenetic generalized linear models) on a large species set, would allow the pattern observed on this study to extend to a broad-scale; excluding the possibility that this pattern is explained by phylogenetic factors other than sexual selection. This is why I suggested a phylogenetic comparative study as a promising future approach, taking advantage of data on a higher number of species and of a resolved phylogenetic tree.

I have read this submission. I believe that I have an appropriate level of expertise to confirm that it is of an acceptable scientific standard, however I have significant reservations, as outlined above.

# Response

**Published:** 2013-09-23

**Authors:** David Bierbach

**Affiliations:** Goethe-University of Frankfurt, Germany

Dear reviewer,

Please find our responses to your points below. We have also indicated the parts of the manuscript which were changed accordingly. We hope our revision has dealt appropriately with your points of critique.

 

1. You are right, we did not quantify the degree of sperm competition in the species investigated here; nevertheless, we have reason to believe that “sexual activity” is a correlate of sperm competition risk. For example, in Bierbach *et al.* (2011) we were able to demonstrate that *P. mexicana* males do not respond with “audience effects” to males that focal males perceive as sexually inactive (equally low SCR), while strong reactions were found in response to males they previously perceived as sexually active (high SCR). On the population (or species) level, the likelihood of females receiving sperm from more than one male ought to be a function of male sexual activity, especially because poeciliid populations in nature tend to be female-biased.

1. We have addressed this point in the discussion: “ *Such an experiment could also shed new light on the observed cross-correlation between sexual activity and aggressiveness as well as between sexual activity and deceptive behavior*.”

1. Sure, the adaptive significance of deceptive behavior is linked to sperm being transferred before the rival enters the mating arena. Still, previous studies have demonstrated rapid sperm transfer in poeciliid fishes when males and females are kept together in similar experimental tanks (for example: Riesch *et al.*, 2008 and Nöbel & Witte, 2013). Investigations on wild Trinidadian guppies suggest high rates of transferred sperm even through forced copulations ( Evans *et al.*, 2003). However, we have added the following sentence to the Discussion: “ *Our interpretation assumes that males initially transferred sperm to the preferred female, which could not be determined unambiguously by simply counting copulation attempts. We thus recommend future experiments that will extract and quantify the amount of transferred sperm from females after the first preference test*".

In response to the minor comment; we added the following half-sentence to the discussion, “ *for example through phylogenetically adjusted generalized linear models on a larger set of poeciliid species*”. However, we have never stated that excluding the feral guppy population will provide phylogenetically independent data.

# Peer review: Referee response for version 2

**Published:** 2013-08-30

**Authors:** Katja Heubel

**Affiliations:** 1 Department of Biology, Institute for Evolution and Ecology, Animal Evolutionary Ecology, University of Tuebingen, Tuebingen, Germany

In the current version of the article, the authors dealt with most of the issues and concerns addressed by the referees.

I have read this submission. I believe that I have an appropriate level of expertise to confirm that it is of an acceptable scientific standard.

# Peer review: Referee response for version 2

**Published:** 2013-08-23

**Authors:** Clelia Gasparini

**Affiliations:** 1 Centre for Evolutionary Biology, The University of Western Australia, Crawley, WA, Australia

The manuscript was already interesting and well written in its first version and the revised version now includes/clarifies the unclear points. I am satisfied with the new version and authors’ replies to my specific comments, and I feel overall that the revisions have resulted in a very interesting paper.

I have read this submission. I believe that I have an appropriate level of expertise to confirm that it is of an acceptable scientific standard.

# Peer review: Referee response for version 1

**Published:** 2013-07-16

**Authors:** Clelia Gasparini

**Affiliations:** 1 Centre for Evolutionary Biology, The University of Western Australia, Crawley, WA, Australia

The authors present a study in which audience effects on male mating behaviour was analysed in several species of poeciliids (a family of livebearing freshwater fish) and related to mean sexual activity (used as a proxy for sperm competition risk) and aggressiveness. This is an attempt to study if sperm competition risk (SCR) can explain the occurrence of audience effects on male choice in this family. The rationale behind this is that males should adjust their mating behaviour by modulating, or even reversing, their initial mate choice in the presence of a rival. The change in male mate choice in the presence of another male has been mainly interpreted as a deceptive signal to lead competitors away from the preferred females, therefore lowering sperm competition risk. Given the complexity of factors (abiotic or biotic) that can contribute simultaneously to shape male mating decisions, explanations other than SCR (though not necessarily mutually exclusive) are also possible, although SCR is certainly likely to be important. Indeed, sperm competition is pervasive in poeciliids, and it is therefore likely that sperm competition is a major force in shaping the evolution of male mating strategies in this family. The hypothesis tested in this paper is that a higher sperm competition risk (SCR) should positively correlate with stronger audience effects across different species. Aggressiveness was also considered, as males could adjust their mate choice to avoid aggressive rivals. 

This is a well written paper, addressing an interesting topic in evolutionary biology. Unfortunately, as the study is only correlative and phylogeny was not accounted for, results can only suggest a general trend, but this can certainly set the stage for future work in this area.No data was collected or analysed to directly quantify SCR in the different species, but total sexual activity (measured in the initial test) was used as a proxy.

Aggressiveness tests:  

The authors performed aggressiveness tests, controlling for a number of factors that can possibly confound interpretation of results, for example, choosing males from different tanks to prevent previously established dominance. However, would aggressiveness scores differ when males are tested in the presence of a female during these encounters? Indeed, two males may have a lot more reasons to exhibit aggressive behaviour when a potential partner is present.

Male mate choice tests:  

In these tests the focal male and two females were free to interact. Methods are described in detail, but I wonder if this is the exact protocol used in all experiments. I am guessing that the method used is probably similar across experiments, but it seems unlikely to me that it is exactly as described here for all of them. Authors also exclude courtship from the sexual activity variable because this behaviour is not present in all species. However, courtship is an important component of sexual behaviour in some of the species considered and including this aspect of male behaviour may therefore change results. 

Main conclusions: 

The main finding that lead the authors to support the hypothesis “SCR is a driving force behind the evolution of this behaviour” is the positive correlation (depicted in fig 3e) between the intensity of sexual behaviour (proxy for SCR) recorded in the first test and the level (occurrence) of deceptive behaviour (the fraction of males that reverse their first choice, based on the first interaction with female, page 7). I would like to know how well the first sexual interaction reflects a male’s sexual choice in these species; is there any direct evidence? In guppies, for example, researchers have tested whether the time spent in front of a female during a binary dichotomous test is a good predictor of actual mating preference (Jeswiet & Godin 2011). Are there any studies that show that first sexual interaction is a reliable sign of male sexual interest in most of the species considered here?

I have read this submission. I believe that I have an appropriate level of expertise to confirm that it is of an acceptable scientific standard, however I have significant reservations, as outlined above.

# Response

**Published:** 2013-08-12

**Authors:** David Bierbach

**Affiliations:** Goethe-University of Frankfurt, Germany

Thank you very much for the positive view of our paper! The reviewer is right, we did not account for phylogeny in our current paper and the main focus of our study was to provide a general comparison of Poeciliid male reproductive behavior given the strong SCR assumed in this family. 

Aggressiveness tests: Your assumptions might be right; in Siamese fighting fish effects of by-standing females on male aggressive behavior has been found (see work by McGregor). However, a recent study showed that Atlantic molly females did not prefer males after they had won a fight ( Bierbach *et al.* 2013) which could lead to reduced aggressive behavior between males when being observed by a female in Poeciliids. 

Male mate choice tests: The described experimental setup was exactly the same in all studies from which we extracted the mate choice data. The reviewer is right, courtship is an important aspect of some of the investigated species’ sexual behavior. Nevertheless, in order to draw general conclusions across a wide range of Poeciliid species that differ in several behavioral and ecological aspects, we focused on sexual behaviors that are directly linked to copulations (thus sperm transfer). Surely, courtship is an aspect that should be investigated in future studies.

Main conclusions: Thank you for this comment! To show that first sexual interactions and mating preferences are congruent, we added another paragraph to the methods section where we explain that in 76%-100% of the trials the first approached females were also subject to the majority of males’ sexual behaviors makes the first sexual approach a good proxy for male preference in all species examined.

# Peer review: Referee response for version 1

**Published:** 2013-06-17

**Authors:** Lisa Locatello

**Affiliations:** 1 Evolution and Ecology of Fish Reproduction, Department of Biology, University of Padova, Padova, Italy

Bierbach and co-authors investigated the topic of the evolution of the audience effect in live bearing fishes, by applying a comparative method. They specifically focused on the hypothesis that sperm competition risk, arising from male mate choice copying, and avoidance of aggressive interactions play a key role in driving the evolution of audience-induced changes in male mate choice behavior. The authors found support to their hypothesis of an influence of SCR on the evolution of deceptive behavior as their findings at species level showed a positive correlation between mean sexual activity and the occurrence of deceptive behavior. Moreover, they found a positive correlation between mean aggressiveness and sexual activity but they did not detect a relationship between aggressiveness and audience effects. 

The manuscript is certainly well written and attractive, but I have some major concerns on the data analyses that prevent me to endorse its acceptance at the present stage.

I see three main problems with the statistics that could have led to potentially wrong results and, thus, to completely misleading conclusions. 

•    First of all the Authors cannot run an ANCOVA in which there is a significant interaction between factor and covariate Tab. 2 (a). Indeed, when the assumption of common slopes is violated (as in their case), all other significant terms are meaningless. They might want to consider alternative statistical procedures, e.g. Johnson—Neyman method. 

•    Second, the Authors cannot retain into the model a non significant interaction term, as this may affect estimations for the factors Tab. 2 (d). They need to remove the species x treatment interaction (as they did for other non significant terms, see top left of the same page 7). 

•    The third problem I see regards all the GLMs in which species are compared. Authors entered the 'species' level as fixed factor when species are clearly a random factor. Entering species as fixed factors has the effect of badly inflating the denominator degrees of freedom, making authors’ conclusions far too permissive. They should, instead, use mixed LMs, in which species are the random factor. They should also take care that the degrees of freedom are approximately equal to the number of species (not the number of trials). To do so, they can enter as random factor the interaction between treatment and species. 

Data need to be re-analyzed relying on the proper statistical procedures to confirm results and conclusions.

A more theoretical objection to the authors’ interpretation of results (supposing that results will be confirmed by the new analyses) could emerge from the idea that male success in mating with the preferred female may reduce the probability of immediate female’s re-mating, and thus reduce the risk of sperm competition on the short term. As a consequence, it may be not beneficial to significantly increase the risk of losing a high quality and inseminated female for a cost that will not be paid with certainty. The authors might want to consider also this for discussion. 

Lastly, I think that the scenario generated from comparative studies at species level may be explained by phylogenetic factors other than sexual selection. Only the inclusion of phylogeny, that allow to account for the shared history among species, into data analyses can lead to unequivocal adaptive explanations for the observed patterns. I see the difficulty in doing this with few species, as it is the case of the present study, but I would suggest the Authors to consider also this future perspective. Moreover, a phylogenetic comparative study would be aided by the recent development of a well-resolved phylogenetic tree for the genus Poecilia (Meredith 2011).

Minor comments:

Page 3: the authors should specify that also part of data on male aggressiveness (3 species from Table 1) come from previous studies, as they do for data on deceptive male mating behavior.

Page 5: since data on mate choice come from other studies is it so necessary to report a detailed description of methods for this section? Maybe the authors could refer to the already published methods and only give a brief additional description.

Page 6: how do the authors explain the complete absence of aggressive displays between the focal male and the audience male during the mate choice experiments? This sounds curious if considering that in all the examined species aggressive behaviors and dominance establishment are always observed during dyadic encounters.

I have read this submission. I believe that I have an appropriate level of expertise to confirm that it is of an acceptable scientific standard, however I have significant reservations, as outlined above.

# Response

**Published:** 2013-08-12

**Authors:** David Bierbach

**Affiliations:** Goethe-University of Frankfurt, Germany

Thank you very much for your overall positive view! In the revised version of our manuscript we rigorously tried to answer all your questions and clear up all points of critique raised. We analyzed our data with the recommended mixed model approach and added a PCA that depicts the species’ behavioral characteristics. 

Statistics: We re-analyzed all data using mixed models with “species ID” included as a subject grouping factor and random intercepts for each species. We then established whether there was significant between-species variation through likelihood ratio tests (model with random intercepts for species vs. reduced model). In the new analysis, random slopes for ‘opponent size difference’ were included for each species in our model so that the final analysis appropriately accounts for species-specific reactions towards this covariate. In the new analysis only significant interaction terms and covariates were retained. For the analysis of the changes in mating preferences (linear mixed model) as well as changed first interactions (generalized linear mixed model) we included ‘treatment’ as a random factor as suggested. 

“ *male success in mating with the preferred female may reduce the probability of immediate female’s re-mating*”: This idea opposes our assumption of general “male mate choice copying” which renders recently mated females more attractive to rivals. If we understand correctly, you suggest some kind of “mate guarding” that would delay re-mating. This is however not a feature of any Poeciliidae mating system known so far. In this context, we would like to refer to our paragraph in the discussion dealing with patterns of sperm precedence in Poeciliids. Up to now, last male sperm precedence is at least verified in one of the species investigated here (for guppies) but it was not the focus of investigations into the other species. Thus, as audience-induced changes in preferences are found in all but one species (namely *H. milleri*), we assume the occurrence of last male sperm precedence is one cause that renders the “risk of losing a high quality and inseminated female” beneficial. 

Phylogenetics: Phylogenetic analysis may be useful, and we re-ran our analysis while excluding the population of feral guppies that were most closely related to the Venezuelan guppies. However, the results remained unchanged. Furthermore, the new PCA that includes all behaviors investigated in the current study does not show any phylogenetic grouping. 

Minor comments: The reviewer is right, the protocol for the mate choice tests as well as the aggression tests are already published but we would like to keep it in the current manuscript for reasons of clarity (also taking advantage of the less restrictive word limits of an online-only journal). In our mate choice tests, focal and audience males were separated as the audience males were fixed in a Plexiglas cylinder. Thus, direct aggression was not observable. Furthermore, a recent study showed that Atlantic molly females did not prefer males after they had won a fight which could have resulted in focal males showing low aggressiveness in front of the two female stimulus fish ( Bierbach *et al. *2013)

# Peer review: Referee response for version 1

**Published:** 2013-06-17

**Authors:** Katja Heubel

**Affiliations:** 1 Department of Biology, Institute for Evolution and Ecology, Animal Evolutionary Ecology, University of Tuebingen, Tuebingen, Germany

I very much appreciate the effort of putting results together to compile a dataset that makes comparison across taxa possible. The authors could take more advantage of that. See more detailed comments below. Unfortunately, the manuscript does not have line numbers.

**Title:** Your title does not really suit your story. In particular, I am not in favour of your Casanova analogy. It is not clear to me how and in which part of your paper you may have tested the males properties of being “Casanovas” – I would have expected you to test a males multiple mating success – which you did not. Furthermore, the “liar” is not clear to me either. You did not test how male’s fitness correlated traits are dishonest or something like this. Anyways, analogies from the liberal arts always come with the problem of being a bit inaccurate since the definition of such characters are always somewhat blurred (is a “Casanova” a male with many matings? Many mates? High reproductive success? Is also male-male competitive ability part of the syndrome? What about the male aggression level?). I suggest refraining from using such non-scientific terms in your title and rather find a suitable title. Reading the abstract and introduction would suggest a title on sperm competition risk and audience effect.

**Abstract:** your start is a bit odd. You start with what seems to me to be the most exceptional example for the cause of sperm competition. Mate copying and even more specific male mate copying is not required for sperm completion. A simple scenario of multiple mating in a promiscuous mating system is a much more straightforward explanation.

**Article content:** the methods section is well explained. Looking at Table 1, I started wondering how many cases were excluded because a clear winner-loser situation could not be established. Comparing N dyads and the df’s, it seems that some species ( *H. milleri*, *P. reticulata feral, L. tridens, P. latipunctata*) have more difficulties in establishing hierarchies under the given size differences. In the mentioned species, no more than 50% of trials ended with a winner-loser situation ( *H. milleri* 4 out of 14 dyads). Thus, the n of trials with established hierarchy is 103, not 146.

Your experimental procedure on male mating behaviour comes with the side-effect that some of the males (and females) got to mate successfully in the first round, but others maybe not. Does this potentially affect their behaviour in front of an audience?

I was wondering whether there would be an alternative and more efficient approach to test species level regressions/correlations? Maybe you can compare regression slopes. Figure 2c may suggest that real deception only occurs in *L. tridens, P. mex. lim, P. mex sulf*, and *P. mex mex* which have an average likelihood to swap preferences and approach the other female of more than 50%.

**Introduction:** the introduction is a bit weak in underlining the novelty and achievements of the current study compared to the already existing body of articles on SCR and audience effects in poeciliids. A reader skimming through the paper may wonder why another study on SCR and audience in poecillids would be an interesting read.

I very much appreciate the effort of putting results together to compile a dataset that makes comparison across taxa possible. Take more advantage of that.

In particular, I was wondering how encounter rates and population density may affect SCR and related coping styles. A life-history approach by looking at shifts in the reproductive periods may be interesting. Depending on whether first or last males sperm precedence is applicable, one may predict an advancement of the mating activity on the population level. Nevertheless, I am aware that this is beyond the scope of this paper.

**Discussion:** I am not convinced that your first sentence is supported by your data: where do you show that variation in audience effects is less pronounced among taxa? Why is personality and behavioural syndromes not touched upon in your introduction? Not sure it really belongs to your story. There is no real data on SCR in your paper. Your introduction deals with SCR in great detail, but it is not really in your data. Is there any solid data that supports your proposed link between sexual activity and SCR?

Data: having 13 taxa at hand, it would be interesting to see which and how some species cluster together. Could you include multiple contrasts or a factor analysis to illustrate similarity vs dissimilarity among species?

As it is, Table 2 with “species” being significant, only reports that at least one species is different from the others. Would be useful to add more information. What you really want to show is how the species are clustering and how this relates to their mating system and sperm competition risk.

I have read this submission. I believe that I have an appropriate level of expertise to confirm that it is of an acceptable scientific standard, however I have significant reservations, as outlined above.

# Response

**Published:** 2013-08-12

**Authors:** David Bierbach

**Affiliations:** Goethe-University of Frankfurt, Germany

Thank you for your positive view of our comparative approach! We tried to outline this cross-taxa character in more detail in the new version of the manuscript. I am afraid the lack of line numbers is a feature of the journal’s publishing and editing concept, sorry. 

You are right to be wary of analogies from the liberal arts. However, the Oxford dictionary defines a “Casanova” as a man notorious for seducing women. As the most sexually active species in our study readily switch their preferred females, we believe that referring to those males as “Casanovas” is not far from reality. The second part of our title adequately describes our current study from a scientific point of view, also incorporating sperm competition as one of your suggested key words. 

Abstract: Thank you for this point! We changed the beginning of the abstract, several parts of the introduction and discussion, as well as the statistical analysis to underpin the comparative approach more precisely.  

Article content: The reviewer is right about the number of dyads in which a clear dominance hierarchy was established. Nevertheless, even when no dominance was established we analyzed the number of aggressive behaviors that occurred and counted those trials as successful. We now precisely state how many trials dominance was established in. 

The reviewer is right, successful and unsuccessful matings could have influenced the behavior in front of an audience. However, in most Poeciliids it is not possible to discriminate with certainty between a successful mating (defined as a mating with sperm being transferred) and the pure mating attempt. We followed our analysis with a protocol from Hammond-Tooke *et al.* (2012) where Spearman’s rank order tests were used to test for behavioral syndromes. We also depict results from PCA to cluster species based on their behaviors (see below). The new analysis found significant between-species variation in the level of deceptive behavior while the general tendency to swap more often between females when confronted with an audience male was found in all but one species. Our current analysis is focused on general behaviors across Poeciliids, more species-based analysis can be found in Bierbach *et al. *(2013) (in press). 

Discussion: Our new analysis shows that between-species variation is much smaller in the audience behavior. We have switched the paragraph dealing with personality and behavioral syndromes from the discussion to the introduction so that the reader will be introduced to these considerations at the beginning. Several studies used the presence of a by-standing rival during mate choice to simulate SCR (see introduction) and sexual activity as a proxy for sperm competition in Poeciliids with high rates of multiple matings (which in turn leads to broods normally sired by multiple males). However, quantifying direct sperm competition (e.g., through offspring genotyping) was beyond the scope of our study. 

Data: To provide an overview about similarity vs. dissimilarity among species we now provide results from PCA.
